# Monoclonal immunoglobulin heavy chain gene rearrangement in Fuchs’ uveitis

**DOI:** 10.1186/s12886-018-0740-3

**Published:** 2018-03-09

**Authors:** Hisae Nakahara, Toshikatsu Kaburaki, Rie Tanaka, Junko Matsuda, Mitsuko Takamoto, Kazuyoshi Ohtomo, Kimiko Okinaga, Keiko Komae, Jiro Numaga, Yujiro Fujino, Makoto Aihara

**Affiliations:** 1 Department of Ophthalmology, University of Tokyo Graduate School of Medicine, 7-3-1 Hongo, Bunkyo-ku, Tokyo, 113-8655 Japan; 2 Department of Ophthalmology, Nerima Hikarigaoka Hospital, 2-11-1 Hikarigaoka, Nerima-ku, Tokyo, 179-0072 Japan; 3 Department of Ophthalmology, Tokyo Metropolitan Geriatric Hospital and Institute of Gerontology, 35-2 Sakae-cho, Itabashi-ku, Tokyo, 173-0015 Japan; 4 Department of Ophthalmology, Japan Community Healthcare Organization Tokyo Shinjuku Medical Center, 5-1 Tsukudo-cho, Shinjuku-ku, Tokyo, 162-8541 Japan

**Keywords:** Fuchs’ uveitis, IgH gene rearrangement, Intraocular lymphoma, Vitrectomy, Vitreous opacity

## Abstract

**Background:**

Fuchs’ uveitis (FU) is occasionarlly complicated with heavy vitreous opacity. We have performed vitrectomy procedures to remove vitreous opacity in affected patients as part of differential diagnosis for primary vitreoretinal lymphoma (PVRL).

**Case presentation:**

We retrospectively reviewed the clinical records of five patients who first visited the Uveitis Clinic of the University of Tokyo Hospital between 2009 and 2013, were diagnosed with FU and underwent a vitrectomy for removal of dense vitreous opacity. All were diagnosed as FU by ocular findings and elevation of Goldmann-Witmer coefficient (GWC) value for the rubella virus (RV) antibody. In examinations of the vitreous body, cytological diagnosis, elevation of IL-10/IL-6 ratio, and the kappa/lambda ratio in flow cytometry findings were negative in all cases, whereas monoclonal immunoglobulin heavy chain (IgH) gene rearrangement was positive in 4 cases and negative in 1 case.

**Conclusions:**

Although monoclonal IgH gene rearrangement is thought to be a reliable biomarker for PVRL, a high percentage of vitreous specimens from our FU patients showed pseudo-positive results. Ophthalmologists must take care regarding possible pseudo-positive findings when performing differential diagnosis between FU and PVRL. Combinations of results of cytological diagnosis, IL-10/IL-6 ratio, kappa/lambda ratio, and IgH gene rearrangement may be necessary for a definitive diagnosis of PVRL and differentiation from FU.

## Background

Fuchs’ uveitis (FU) was first reported by Fuchs in 1906 and affected patients are characterized by iris heterochromia, mild iridocyclitis, iris atrophy, and keratitic precipitates [1]. Since the etiology remains unknown, FU is usually determined based on clinical manifestations, because the etiology of FU is still unknown. Many authors used the classical triad of signs of FU, first described by Kimura [2]. However, a diagnosis of FU is sometimes difficult because the various related clinical signs are not always simultaneously present. To specify an international standard for diagnosis, La Hey proposed diagnostic criteria for FU consisting of four essential and seven associated findings [3]. The essential findings of FU are absence of acute symptoms, characteristic keratic precipitates and/or minimal cells and flare in the aqueous, diffuse iris stromal atrophy, and absence of synechiae. Associated findings are unilaterality of uveitis, heterochromia, iris posterior pigment epithelium atorophy, subcapsular cataract, elevated intraocular pressure, vitreous opacity, and chorioretinal lesions. For definitive diagnosis, all essential findings and at least 2 of 7 associated findings must be present.

Recently, several reports have demonstrated a relationship of FU with rubella virus (RV) infection. Intraocular synthesis of the RV antibody was detected in aqueous humor samples from 95 to 100% of FU patients [4–6], while RV-RNA was detected by reverse transcription-polymerase chain reaction (RT-PCR) in those samples from 10 to 22% of the patients with FU [4–6]. Those results suggested that RV is an antigenic stimulus for an oligoclonal B-cell response in most eyes of FU patients, indicating that an examination for the presence of the RV antibody in aqueous humor is useful for diagnosis [5].

On the other hand, vitreous opacity is a characteristic finding of FU. Previous studies have reported detection of vitreous opacity in most (84–97%) examined patients with FU [3, 7], with dense vitreous opacity seen in 15% of those in the report [7]. In another, it was noted that a vitrectomy for removal of dense vitreous opacity was necessary in 8% of patients with FU [8]. Since vitreous opacity is one of the common characteristic features of primary vitreoretinal lymphoma (PVRL), FU is one of the disease recommended be made differential diagnosis [9]. Although detection of malignant cells in cytology findings is the gold standard for diagnosis of PVRL, the false-negative rate has been reported to range from 30 to 45% [10, 11]. Therefore, examinations of vitreous humor samples, such as immunohistochemistry of slide-mounted cells [12], IL-10/IL-6 ratio (> 1) [10, 13], kappa/lambda ratio (> 3 or < 0.6) shown by flow cytometry [12], and immunoglobulin heavy chain (IgH) gene rearrangement, are recommended for facilitating an accurate diagnosis [13]. However, even though ocular findings of FU can be similar to those of PVRL, no reports regarding examinations of vitreous samples from FU patients have been presented.

In the present study, we retrospectively investigated findings of vitreous samples obtained from 5 FU patients, each of whom was definitely diagnosed based on results showing intraocular synthesis of the RV antibody. To clarify factors for differential diagnosis between FU and PVRL, we noted the results of cytology, immunohistochemistry on slide-mounted cells, IL-10/IL-6 ratio, kappa/lambda ratio by flow cytometry, and IgH gene rearrangement in these cases.

## Case presentation

We obtained the clinical records of five patients (5 eyes; 4 males, 1 female) who first visited the Uveitis Clinic of the University of Tokyo Hospital between 2009 and 2013 and were diagnosed with FU based on ocular manifestations [3]. Each underwent a vitrectomy procedure for removal of dense vitreous opacity. The mean follow-up period was 20.4 ± 19.2 months (range 3.8-51.5 months). Whereas three patients had rubella infection history, two patients didn’t have rubella infection history clearly. All five patients and their mothers didn’t receive rubella vaccination, because the vaccination of rubella had only performed between 1988 and 1993 and it had stopped because of vaccination induced encephalitis.

The surgical procedure we adopted was core vitrectomy by 20 Gauge or 23 Gauge. Peripheral vitrectomy was not completely performed. Vitreous specimens from the present patients were examined for determination of RV antibody titer and total IgG in vitreous humor and serum to determine the Goldmann-Witmer coefficient (GWC) [14] value for the RV antibody, IL-6 and IL-10 concentrations, cytological diagnosis based on Papanicolou staining and immunohistochemistry results, kappa/lambda ratio based on flow cytometry results, and IgH gene rearrangement.

As for cytology, undiluted vitreous samples were immediately fixed by 10% formalin and prepared for cytology with Papanicolou staining. As for rubella antibody titer, total IgG and the concentrations of IL-10 and IL-6, undiluted vitreous specimens were centrifuged and the concentrations of rubella antibody titer, total IgG, IL-10 and IL-6 in the supernatants were measured by ELISA (SRL, Inc. Tokyo, Japan). As for the IgH gene rearrangement, 10 ml of diluted vitreous samples were centrifuged and the pellet were obtained. DNA was extracted from the pellets and used for the analysis of IgH gene rearrangements (LSI Medience Corporation, Tokyo, Japan) [15]. As for flow cytometry, vitreous bodies were digested in a 1 mg/ml solution of collagenase type II in standard culture medium with shaking at 37 °C for 1 h. After the extracted cells in the vitreous body were washed once with sterile PBS, the cells were analyzed with a staining of fluorescein isothiocyanate conjugated anti-IgG kappa light chain and phycoerythrin conjugated anti-IgG lambda light chain using a FACS Aria Flow Cytometer (BD Biosciences, Heidelberg, Germany). As for the diagnosis of PVRL, the positive rates of cytology in the vitreous samples (class 4 or more) had been reported to be quite low (44.5%) [10]. Therefore, combination of cytology and supplemental cytokine analysis and molecular analysis should be recommended to facilitate the diagnosis of intraocular lymphoma [9, 10, 13]. In our hospital, vitreous samples were examined for cytology, IL-10/IL-6 ratio, FACS (kappa/lambda), and IgH gene rearrangement. PVRL was determined by positive cytology findings of a vitreous sample (class 4 or more) or positive results for more than 2 of the following 4 items: class 3 cytology, IL-10/IL-6 concentration ratio > 1, positive IgH rearrangement, and light chain restriction shown by flow cytometry (kappa/lambda ratio > 3 or < 0.5) [16].

A summary of ocular findings in the present 5 FU cases are shown in Table 1. All fulfilled the diagnostic criteria of FU presented by La Hey [3]. One patient (case 3) underwent a vitrectomy procedure in combination with cataract surgery and four patients underwent vitrectomy procedure for vitreous opacity (Table 2). As for best-corrected visual acuity (BCVA), a post-operative increase of BCVA (2 grades or more) was observed in 1 (case 4), while that was decreased (2 grades or more) in 1 (case 3). In case 3, BCVA was decreased from 0.8 before the operation to 0.5 after, likely due to post-operative macula atrophy. In all cases, floaters were removed by the vitrectomy procedure.

**Table 1:** Summary of ocular findings in five patients with Fuchs’ uveitis

			Essential findings				Associated findings						
Patient no.	Age (yrs)	Sex	Absence of acute symptoms	Characteristic keratic precipitates and/or minimal cells and flare in aqueous	Diffuse iris stromal atrophy	Absence of synechiae	Unilaterality of uveitis	Heterochromia	Iris posterior pigment epithelium atrophy	Subcapsular cataract	Elevated intraocular pressure	Vitreous opacity	Chorioretinal lesions
1	35	M	(+)	(+)	(+)	(+)	(+)	(−)	(−)	(−)	(−)	(+)	(−)
2	29	F	(+)	(+)	(+)	(+)	(+)	(−)	(−)	(+)	(−)	(+)	(−)
3	57	M	(+)	(+)	(+)	(+)	(+)	(+)	(−)	(+)	(−)	(+)	(−)
4	51	M	(+)	(+)	(+)	(+)	(+)	(+)	(−)	(−)	(−)	(+)	(−)
5	54	M	(+)	(+)	(+)	(+)	(+)	(+)	(−)	(+)	(−)	(+)	(−)

*M* male, *F* female

**Table 2:** Summary of outcomes of vitrectomy for vitreous opacity in Fuchs’ uveitis patients

Patient no.	Operation	BCVA (pre-operation)	BCVA (latest visit)	Complications
1	PPV	1.2	1.2	(−)
2	PPV	1.0	1.0	Retinal tear (during operation)
3	PPV + PEA+IOL	0.8	0.5	Macula atrophy (after operation)
4	PPV	0.6	1.0	(−)
5	PPV	1.0	1.2	(−)

*BCVA* best-corrected visual acuity, *PPV* pars plana vitrectomy, *PEA* phacoemulsification and aspiration, *IOL* intraocular lens

Results of examinations of the vitreous specimens are shown in Table 3. The RV antibody was detected in both serum and vitreous specimens in all cases. Moreover, the GWC value for RV was calculated to be greater than 5 in all cases, providing definitive evidence that these cases were FU [5, 6]. On the other hand, the IL-10/IL-6 ratio was less than 1 and cytological diagnosis was class 2 or lower in all 5 cases. As for flow cytometry, the T-cell (CD3)/B-cell (CD19) ratio was greater than 1 in 3 cases and undetermined due to insufficient samples in the other 2. Our result of more T cells than B cells in vitreous body of FU is in agreement with previous report [17, 18]. The kappa/lambda ratio was within normal limits (≥0.6 and ≤3) in 4 cases and could not be determined due to an insufficient sample in 1. These results of all cases were not in agreement with PVRL (Table 3). As for IgH gene rearrangement, 4 of 5 cases were positive and only 1 case was negative, indicating that IgH gene rearrangement findings can lead to pseudo-positive findings in examinations of vitreous samples to differentiate FU from PVRL.

**Table 3:** Summary of examinations of vitreous specimens from patients with Fuchs’ uveitis

Patient no.	Rubella antibody titer in blood (HI method)	Rubella antibody titer in vitreous body (HI method)	Goldmann Witmer coefficient	IL-10 (pg/ml)	IL-6 (pg/ml)	IL-10/IL-6	Cytology class	Flow cytometry	κ/λ	IgH rearrangement
1	128×	16×	> 5.0	< 2	29.8	< 1	2	T-cell > B-cell	1.8	(+)
2	128×	128×	28.6	< 4	42.0	< 1	2	Insufficient sample	0.7	(+)
3	64×	32×	> 13	< 4	45.8	< 1	2	Insufficient sample	Insufficient sample	(+)
4	64×	128×	> 50	< 2	24.4	< 1	1	T-cell > B-cell	2.0	(+)
5	128×	64×	> 11.1	< 8	36.4	< 1	2	T-cell > B-cell	1.3	(−)

*HI* hemagglutination inhibition, *IL* interleukin, *IgH* immunoglobulin heavy chain

## Discussion and conclusions

Recently, many reports had suggested that the GWC for the rubella virus were specifically elevated in patients with FU [4–6]. However, we usually diagnose FU by clinical symptoms even now in clinical practice. Thus, at the current moment, the GWC for rubella virus is used as an adjunct to the diagnosis of FU.

Scott [19] and Waters [20] investigated FU patients who underwent a vitrectomy for removal of dense vitreous opacity and reported that at least a 2 grade increase in BCVA was obtained in about 70% of those patients, while floaters were removed in nearly all of their cases. In the present study, an increase in post-operative BCVA of 2 grades or more was observed in only a single patient, which might have been due to the excellent pre-operative BCVA findings in our case series.

In examinations of vitreous samples for differential diagnosis between FU and PVRL, cytological diagnosis, IL-10/IL-6 ratio, and kappa/lambda ratio shown by flow cytometry were all negative in our five patients. The predominance of T-cells over B-cells in vitreous samples from FU patients has been reported [17], and was also seen in the present cases. Unexpectedly, IgH gene rearrangement was positive in 4 of 5 cases of our FU patients, each of whom were definitively diagnosed based on confirmation of a GWC value for the RV antibody of greater than 5. IgH gene rearrangement has been reported to be a reliable biomarker for PVRL [13]. However, this result clearly demonstrated that IgH gene rearrangement can be a pseudo-positive finding in vitreous samples from FU patients. Actually, the possibility of FU accompanied with PVRL in those patients could be conceivable for the explanation of the current results. However, previous studies have reported that intracranial lymphoma develops in 60–85% of patients with PVRL, usually within 29 months, when medical treatment was not performed [21]. And the life prognosis is quite exacerbated by intracranial lymphoma developing [22]. In this study, five patients were followed-up for 20.4 ± 19.2 months (range 3.8-51.5 months) and no one suffered intracranial lymphoma. In addition, because Fuchs uveitis and PVRL are both rare disease, the possibility of FU accompanied with PVRL is quite very low. Thus, we believe that the current results of positive IgH gene rearrangements are pseudo-positive.

Another question could be considerable if these examinations must be performed using vitreous samples or could be performed using aqueous humor sample. In this study, all of the examinations were performed using undiluted and diluted vitreous specimens, because many examination items and some quantity of sample volumes must be necessary for the diagnosis of PVRL. The samples from aqueous taps from anterior chamber might not be enough for the analysis of PVRL especially in cytology and FACS. Thus, we believe that the differential diagnosis of FU and PVRL should be performed by vitreous samples obtained by vitrectomy.

A few reports have noted that IgH gene rearrangement can be positive in patients with an infectious disease, especially viral hepatitis. A monoclonal IgH gene rearrangement was detected in 10-32.1% of liver biopsy specimens from hepatitis C virus (HCV)-infected patients [23, 24], and in 0.4% of liver biopsy specimens from hepatitis B virus (HBV)-infected patients [23]. The present finding suggests that FU patients might be complicated with chronic immune reactions against RV and the RV antigen could frequently induce monoclonal B cell expansion in their eyes. Thus, ophthalmologists must take care regarding IgH gene rearrangement as a pseudo-positive finding indicating a diagnosis of PVRL in a patient with FU.

In conclusion, IgH gene rearrangement may be frequently found in vitreous specimens of FU cases. A combination of findings including cytological diagnosis, IL-10/IL-6 ratio, flow cytometry, and IgH gene rearrangement are necessary for an accurate diagnosis of PVRL and differentiation from FU.

## Abbreviations

FU: Fuchs’ uveitis
GWC: Goldmann-Witmer coefficient
HBV: Hepatitis B virus
HCV: Hepatitis C virus
IgH: Immunoglobulin heavy chain
PVRL: Primary vitreoretinal lymphoma
RV: Rubella virus
